# Bioinformatic training needs at a health sciences campus

**DOI:** 10.1371/journal.pone.0179581

**Published:** 2017-06-14

**Authors:** Jeffrey C. Oliver

**Affiliations:** University of Arizona Health Sciences Library, University of Arizona, Tucson, AZ, United States of America; Universidad de las Palmas de Gran Canaria, SPAIN

## Abstract

**Background:**

Health sciences research is increasingly focusing on big data applications, such as genomic technologies and precision medicine, to address key issues in human health. These approaches rely on biological data repositories and bioinformatic analyses, both of which are growing rapidly in size and scope. Libraries play a key role in supporting researchers in navigating these and other information resources.

**Methods:**

With the goal of supporting bioinformatics research in the health sciences, the University of Arizona Health Sciences Library established a Bioinformation program. To shape the support provided by the library, I developed and administered a needs assessment survey to the University of Arizona Health Sciences campus in Tucson, Arizona. The survey was designed to identify the training topics of interest to health sciences researchers and the preferred modes of training.

**Results:**

Survey respondents expressed an interest in a broad array of potential training topics, including "traditional" information seeking as well as interest in analytical training. Of particular interest were training in transcriptomic tools and the use of databases linking genotypes and phenotypes. Staff were most interested in bioinformatics training topics, while faculty were the least interested. Hands-on workshops were significantly preferred over any other mode of training. The University of Arizona Health Sciences Library is meeting those needs through internal programming and external partnerships.

**Conclusion:**

The results of the survey demonstrate a keen interest in a variety of bioinformatic resources; the challenge to the library is how to address those training needs. The mode of support depends largely on library staff expertise in the numerous subject-specific databases and tools. Librarian-led bioinformatic training sessions provide opportunities for engagement with researchers at multiple points of the research life cycle. When training needs exceed library capacity, partnering with intramural and extramural units will be crucial in library support of health sciences bioinformatic research.

## Introduction

The burgeoning fields of bioinformatics and biomedical informatics have produced a wealth of databases and analytical tools important in health sciences research. These resources are creating unprecedented opportunities for discovery, yet they require significant training to fully exploit their utility [1]. In addition to knowledge of biological data repositories, analytical and computational skills have an increasing role in health sciences research [2, 3]. This demand for knowledge and skills has, in turn, created a significant need for training in identification and use of said resources in health sciences research [4–6].

In support of these non-bibliographic information resources, many libraries have bioinformatic or molecular biology programs [7–9], often employing specialists with research backgrounds in the life or health sciences. The University of Arizona Health Sciences Library (UAHSL) recently established a Bioinformation program, dedicating a full-time specialist to facilitate effective bioinformatic research. In addition to providing on-demand services for navigating biological databases and assistance with bioinformatic analyses, the library is tailoring the program to meet the needs of the University of Arizona Health Sciences campus. To this end, I developed a needs assessment survey to better understand the nature of support the library could provide to health sciences researchers. Such an assessment is critical to (1) enumerate the needs of the specific institution, (2) identify those needs for which the library could provide education and training support, and (3) identify needs best met by partnering with units outside the library [9, 10]. Here I present the results of the survey, highlighting areas of interest at the University of Arizona Health Sciences. The efforts taken at the UAHSL to meet these training needs are also discussed as an example of how to integrate the library into bioinformatic research in the health sciences.

## Methods

This survey was designed to assess the bioinformatics training needs of health sciences researchers. Questions were developed based on (1) current trends in bioinformatics, (2) previous assessments of training needs at other institutions [8–12], and (3) feedback from librarians at UAHSL and other institutions. Briefly, the survey included questions about the level of interest in different training topics, preferred training formats, and current use levels of library resources. The survey also included basic demographic questions (e.g. status, affiliation) and questions about current research. A copy of the full survey is available in supplementary information (S1 File).

The two main questions of the survey aimed to assess the “what” and “how” of bioinformatics training. That is, (1) which potential offerings are of primary interest to health sciences researchers; and (2) which training formats are preferred modes of learning for bioinformatics topics. To address the former, participants were presented with an enumeration of twelve general bioinformatics topics and asked to indicate their interest level in each topic. Questions were presented as Likert items, with 1 indicating “not interested” and 5 indicating “very interested” [8]. To determine preferred mode of training, participants were presented with a list of six training methods (hands-on workshop, lecture, one-on-one session, group session, online tutorial, and webinar) and asked to indicate which methods were preferred.

### Administration

The survey was administered online and participants were solicited by direct e-mail. Invitations to participate were e-mailed to all 809 graduate students, faculty, and staff in the four Tucson colleges of the University of Arizona Health Sciences (UAHS) campus. The survey opened November 17, 2015 and closed on December 16, 2015 (30 days); direct e-mail reminders were sent on days 15 and 28. I used the online Qualtrics (www.qualtrics.com) platform for collecting responses. Results were anonymous, IP addresses were not recorded, and participation in the survey was entirely voluntary. It was determined by University of Arizona IRB members that because the results of the survey are not broadly generalizable, IRB approval was not required.

### Statistics

To test for significant preferences, I first used a Kruskal-Wallis omnibus test for differences among groups; for subsequent tests of preferences where the omnibus test was significant, I used ordinal logistic regression for training interests and logistic regression for training formats. Results were considered significant at the α = 0.05 level, except in cases where multiple tests were performed, in which case a Bonferroni correction was applied. All statistics were performed with the R software statistics package [13], as well as the dplyr [14], tidyr [15], and MASS [16] packages; plots were created using the ggplot2 package [17]. Survey results and R code for all analyses and graphics can be found at (https://github.com/jcoliver/ua-bioinfo-survey).

## Results & discussion

### Demographics

A total of 72 participants completed the survey (~9% of 809 invitees), with the majority of responses (N = 43) coming from the College of Medicine, the largest college at UAHS (Table 1). On average, participants completed the survey in a little under four and a half minutes. The e-mail reminder messages were critical in obtaining responses to the survey: although the survey was open for 30 days, 89% (N = 64) of responses occurred within twelve hours of one of the three e-mail messages about the survey (one announcement and two reminders) (S1 Fig). This marked response to e-mail messaging strongly suggests that reminder frequency has a significant effect on survey participation rates.

**Table 1:** Survey participant demographics (total number of responses: 72).

Position		
	Faculty	33 (45.8%)
	Staff	15 (20.8%)
	Student	21 (29.2%)
	Not provided	3 (4.2%)
College / Unit		
	Arizona Biological and Biomedical Program	1 (1.4%)
	BIO5 Institute	1 (1.4%)
	College of Medicine	44 (61.1%)
	College of Nursing	2 (2.8%)
	College of Pharmacy	7 (9.7%)
	Mel & Enid Zuckerman College of Public Health	6 (8.3%)
	College of Science	5 (6.9%)
	Not provided	7 (9.7%)
Time at University of Arizona		
	0–5 years	31 (43.1%)
	5–10 years	13 (18.1%)
	10–20 years	13 (18.1%)
	>20 years	6 (8.3%)
	Not provided	9 (12.5%)

Percentages in the College / Unit section do not sum to 100% due to dual affiliations of some respondents.

### Training interests

**Fig 1:**
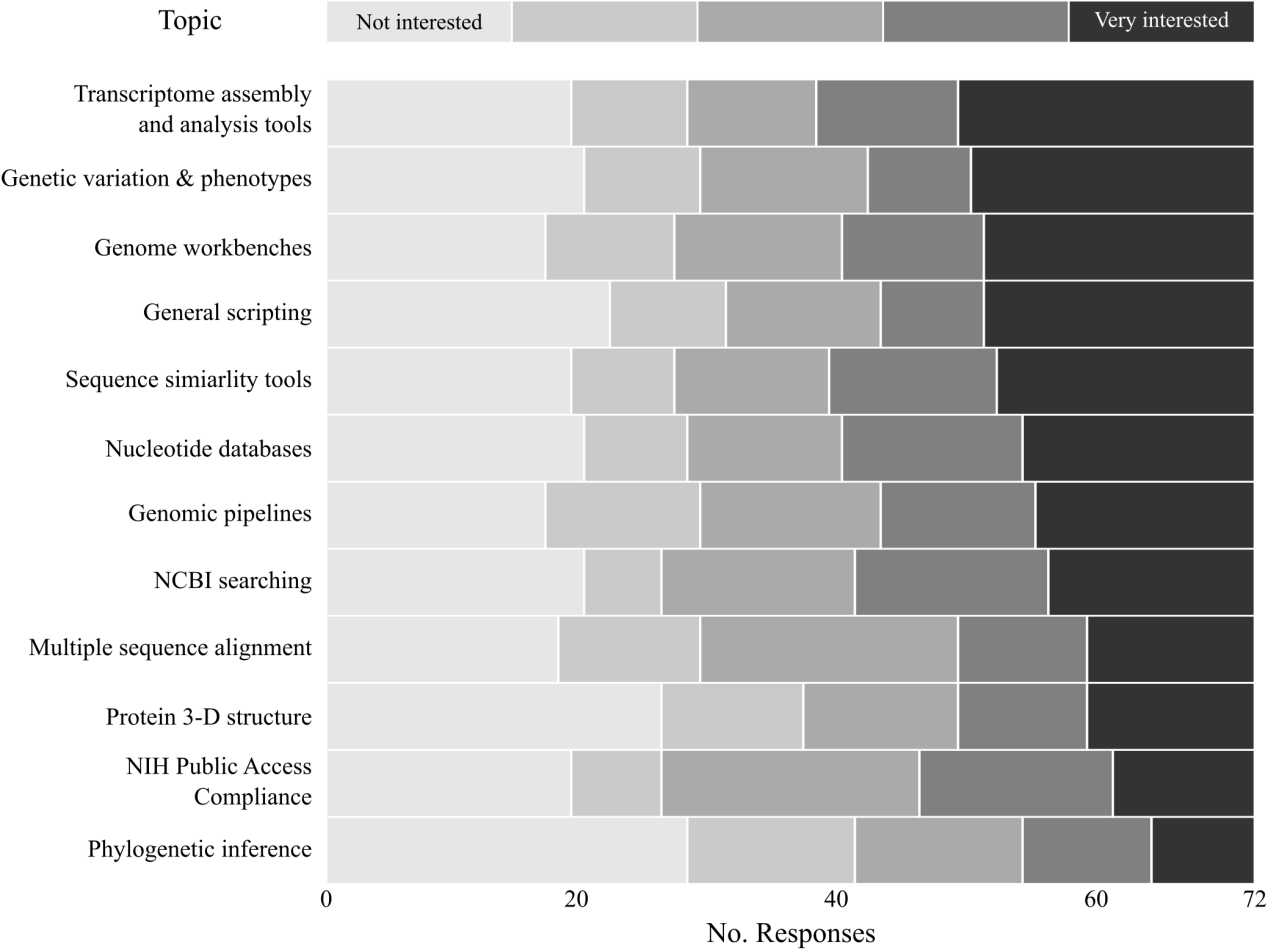
Interest levels in potential bioinformatic training topics.

**Fig 2:**
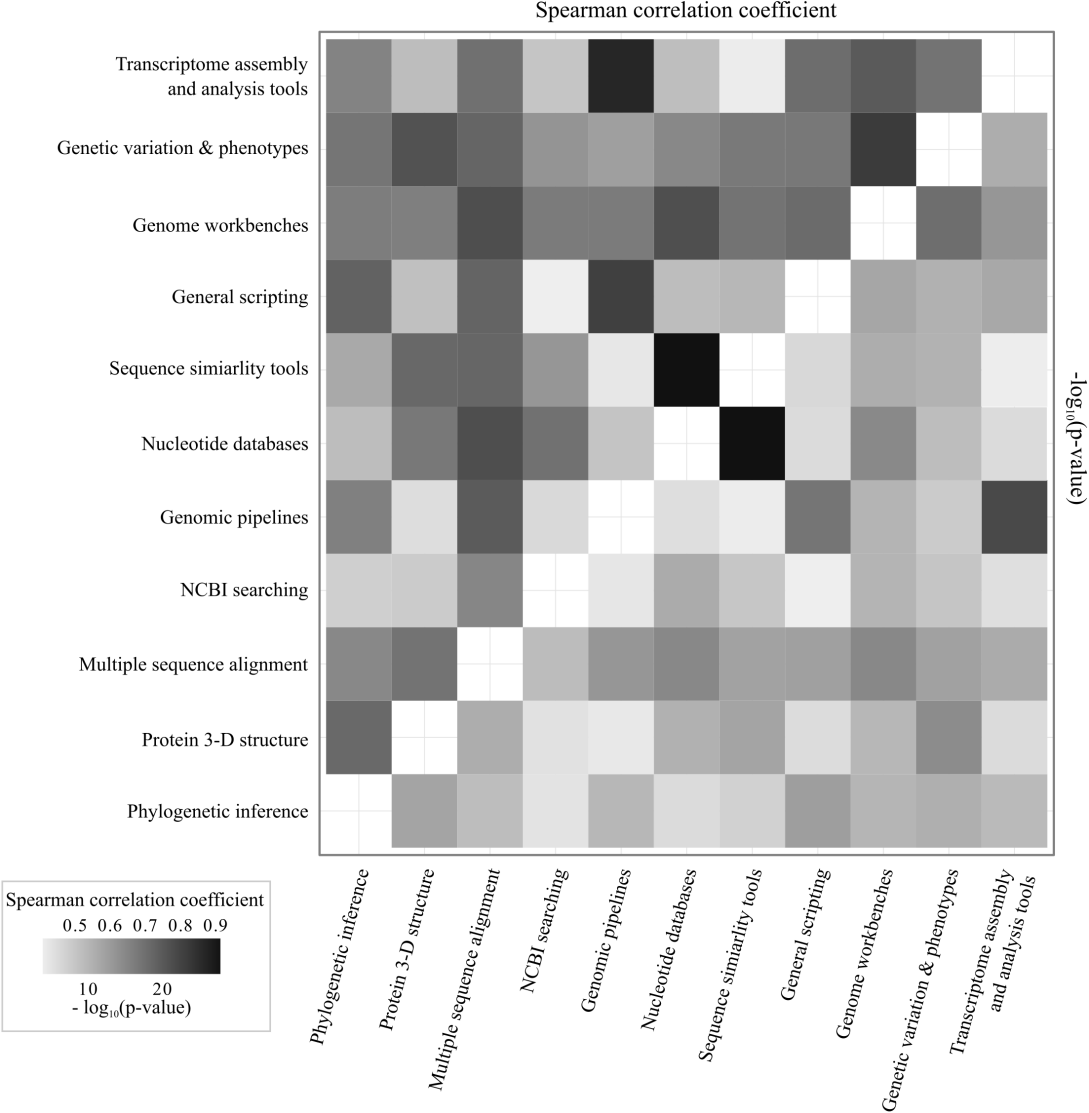
Pairwise comparisons of interest levels in training topics. All correlations significant after Bonferroni adjustment for multiple comparisons (p < 0.00091).

In general, there was a demonstrated interest in bioinformatic training, as 61% (N = 44) of respondents were "very interested" in receiving training in at least one topic and 79% (N = 57) scored an interest level of 4 or 5 for training in at least one topic. Given that participation in the survey was entirely voluntary, there is potential for significant self-selection by researchers who are already interested in bioinformatics training; however, 10% of survey participants (N = 7) responded that they were "not interested" in any of the potential training topics, indicating the respondent pool included some health sciences researchers with little evident interest in bioinformatics training. While all training topics garnered at least some interest (Fig 1), no topics were significantly preferred over others (Kruskal-Wallis χ^2^(11) = 15.952, *p* = 0.1429). There was significant correlation in interest levels between the various topics; i.e. respondents who were "very interested" in one topic were likely to be interested in other topics as well (Fig 2). Following Bonferroni correction for multiple tests, all pairwise comparisons showed significant correlations between preferences, suggesting that some respondents were interested in bioinformatics training overall, while others had little interest in bioinformatics training in general. Notable relationships were correlated preferences for sequence similarity tools (e.g. BLAST [18]) and nucleotide databases (Spearman's *ρ* = 0.91, corrected *p* < 0.001) and correlation between transcriptomic tools and genomic pipelines (Spearman's *ρ* = 0.85, corrected *p* < 0.001).

There were marked differences in interest based on the career stage of the respondent. In an omnibus test, position (faculty, staff, or student) significantly affected the interest level across the various training topics (Kruskal-Wallis χ^2^(2) = 58.621, *p* < 0.001). In *post hoc* comparisons between position types, faculty demonstrated the lowest interest in bioinformatics training across topics, staff demonstrated the highest, and students expressed an intermediate level of interest (Table 2). The "staff" category includes post-doctoral scholars and research scientists, who, among the three groups, may be the most active in bioinformatic research, given the demands of the other two categories. That is, the high interest in bioinformatics training by staff likely reflects the fact that bioinformatic analyses comprise a large majority of their work distribution, while most faculty have teaching and service obligations and students are involved in coursework. While overall training interest did significantly vary among position types, when data for each position were analyzed separately, there did not appear to be significant preferences for particular training topics (faculty: Kruskal-Wallis χ^2^(11) = 7.448, *p* = 0.7617; staff: Kruskal-Wallis χ^2^(11) = 12.705, *p* = 0.3131; student: Kruskal-Wallis χ^2^(11) = 12.669, *p* = 0.3155).

**Table 2:** Differences in interest levels among positions.

Position	Faculty	Staff	Student
Faculty	2.616 (0.078)	< 0.0001	0.001
Staff	7.562	3.672 (0.095)	0.0001
Student	3.302	-4.381	2.996 (0.092)

Results are from *post hoc* logistic regression model, where the position in each row was used as a reference against the remaining two positions. Diagonal values are the mean interest level (SE) across all training topics (on a scale of 1 to 5), *p*-values are shown above the diagonal, and *t* values are below the diagonal.

The two topics with the most respondents answering "very interested" concerned transcriptome analyses and databases focused on genetic variation and phenotypes (Fig 1). The interest in transcriptomics reflects the current high level of interest in the acquisition and analysis of high-throughput gene expression data. While this is an area of keen interest for many researchers in the health sciences field, it requires considerable specialized knowledge that most library staff may not possess. To fulfill training needs in transcriptomics, libraries could facilitate training sessions by local researchers actively using transcriptomic resources or by organizing on-site training sessions by inviting speakers from specific resources (e.g. Galaxy Project, https://wiki.galaxyproject.org/Outreach#Speakers). The interest in training in genotypic and phenotypic variation databases may be easier to address, and a number of resources exist for learning how to use such databases. Multiple databases on genotypes and phenotypes are hosted by the National Center for Biotechnology Information (https://www.ncbi.nlm.nih.gov), and NCBI has produced several instructional videos for using databases (https://www.youtube.com/user/NCBINLM). Another host of genomic and phenotypic data resources is Ensembl (http://www.emsembl.org), which also provides tutorials and written materials that could be used in library-hosted workshops (http://www.ensembl.org/info/website/tutorials/index.html). Finally, the UCSC Genome Browser team offers on-site training tailored to the audience's interest and level of expertise (https://genome.ucsc.edu/training/). The degree to which libraries can fulfill the training needs in transcriptomics and genomic databases using in-house resources will depend largely on librarians' domain knowledge in these areas.

One particular result of note was the distribution of interest level in general scripting training. Respondents were most polarized in their responses to this topic: 60% of respondents were either "not interested" or "very interested" in receiving training in this topic (Fig 1). The polarization is interesting because skills for automating tasks through scripting languages are increasingly important in pursuing bioinformatic research [1, 19]. Indeed, the topic garnering the most interest, transcriptomic analyses, relies heavily on command-line and scripting proficiency. The National Institutes of Health recognized the importance of computational literacy in exploiting big data for biomedical research and established the Big Data to Knowledge (BD2K, https://datascience.nih.gov/bd2k) program in 2012. Part of the BD2K mission is to improve training for health sciences researchers in the use of tools necessary for large-scale biomedical data analyses. The relatively high number of respondents who were interested in this training likely reflects a recognition of the importance of such tools in bioinformatic research; however, general scripting also had the third-most respondents who were "Not interested" in receiving training on the topic. The cause of this disinterest could not be addressed with the current survey, but may partly reflect general computer anxiety, ignorance of the utility of scripting skills, or sufficient programming proficiency by a large number of respondents.

Providing training in general scripting to health science researchers presents a variety of challenges, but none are insurmountable for addressing this key skill set in bioinformatics. Many scientists have little to no programming experience, but teaching basic concepts as system paths and command-line interfaces are deemed "too rudimentary" for many academic computer science departments [20]. Add to this the numerous time constraints on researchers and committing time to a semester- or quarter-long computer programming course becomes difficult to justify. Library staff can address this training need in a variety of ways including: providing training themselves in the format of hands-on workshops (see *Training formats*, below), if they have the background in one or more scripting languages; facilitating training by staff from a campus bioinformatics service center [10]; or organizing one or more Software Carpentry workshops (http://software-carpentry.org/), which provide novice-level instruction for scientists on topics such as the command-line interface, version control, and a scripting language (generally R or python). Library support for entry-level skills training in bioinformatic analytical approaches, including writing computer scripts, presents an opportunity for research libraries to engage with clientèle at another contact point in the research life cycle.

Topics receiving low levels of interest generally involved specialized areas applicable to a limited number of researchers at the UAHS. Training in multiple sequence alignment, protein 3D structure, and phylogenetic analyses all received relatively lower levels of interest. Also of low interest was training in compliance with NIH public access policies, which may reflect a general disinterest in dealing with publication and data sharing mandates.

### Training formats

**Fig 3:**
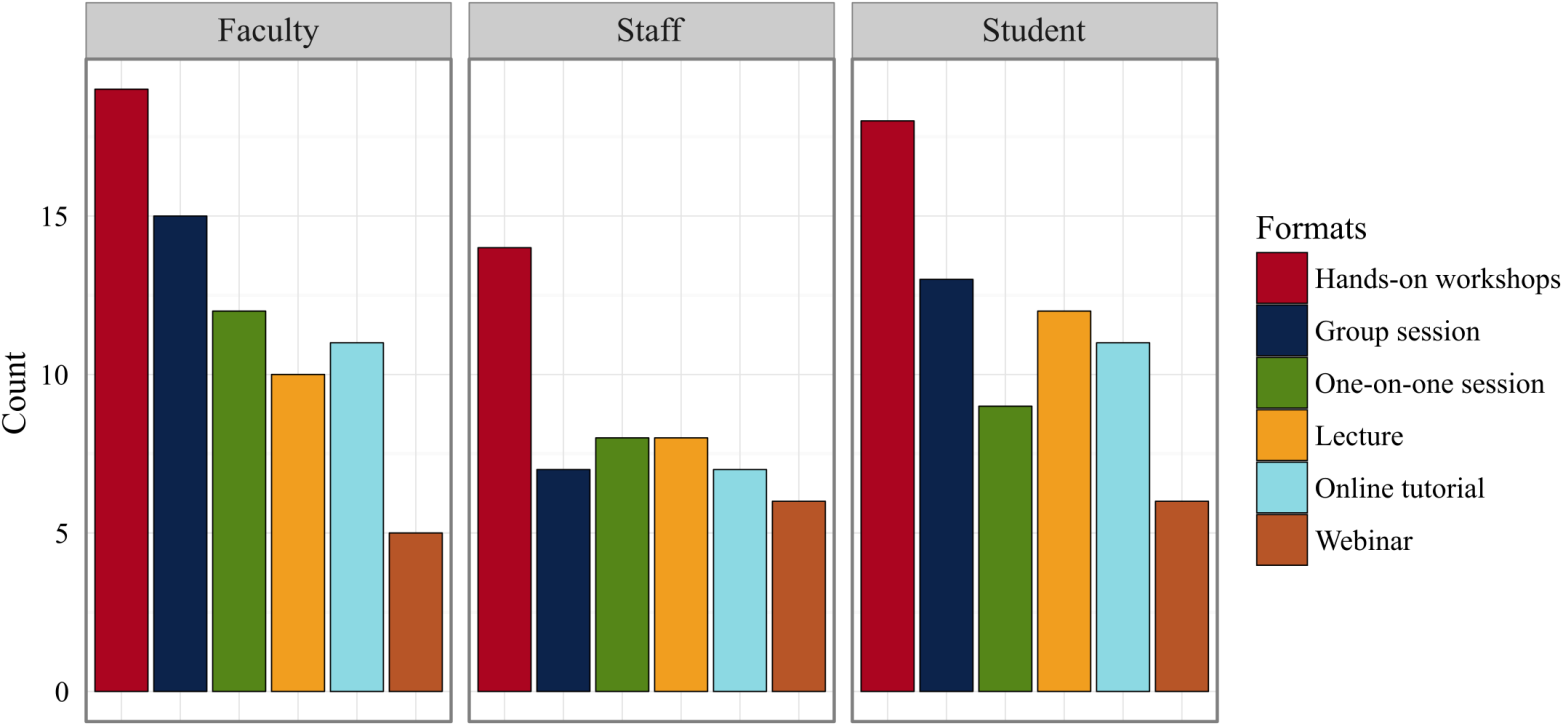
Training format preferences by participants' position.

There were significant differences among preferences for the different training formats in an omnibus test (Kruskal-Wallis χ^2^(5) = 32.901, *p* < 0.001). Hands-on workshops had the most support: 72% (N = 52) of respondents marked it as a preferred format, significantly more than any other workshop format (Table 3, Fig 3). The desire for hands-on skills training mirrors similar preferences reported in previous studies [21, 22]. Via et al. 2011 [23] provide an apt analogy for why workshops are critical in bioinformatics training: "Acquiring [bioinformatics] skills is a bit like learning to ride a bicycle, where it is best to just start pedalling, because watching others will not help you learn the process!" (p. 2).

**Table 3:** Preference for hands-on workshops over other training formats.

Format	Coefficient	Std. Error	*z*	Pr(>|*z*|)
Group session	-0.9786	0.3581	-2.733	0.006274
One-on-one session	-1.3192	0.3600	-3.665	0.000248
Lecture	-1.2617	0.3593	-3.511	0.000446
Online tutorial	-1.2617	0.3593	-3.511	0.000446
Webinar	-2.0136	0.3791	-5.311	1.09e-07

Results are from *post hoc* logistic regression model, where workshop is the reference format.

### Addressing researchers' needs

Bioinformation support at UAHSL is an evolving program, but a number of actions have already been taken to address bioinformatic training needs. First, a number of short, hands-on workshops have been delivered to health and life science researchers, covering bioinformation resources such as genome browsers (NCBI's Variation Viewer, https://ncbi.nlm.nih.gov/variation/view) and online gene expression analysis tools (GEO2R, https://ncbi.nlm.nih.gov/geo/geo2r). An ongoing series of introductory R workshops, designed for scientists with little to no programming experience, are offered by UAHSL on the University of Arizona Health Science campus. In addition to in-house library workshops, the University of Arizona Libraries also partnered with external organizations to provide workshops for health sciences researchers, including the Software Carpentry Foundation (http://software-carpentry.org/) to introduce programming skills for reproducible scientific analyses and the Center for Open Science (https://cos.io) to showcase the Open Science Framework (https://osf.io/) for project management and reproducible science. Finally, several other campus service units that provide bioinformatic support have been identified and enumerated online (http://libguides.library.arizona.edu/bioinfo/campus-services); when researchers' needs extend beyond the library's capacity, they are referred to these other units.

### Challenges & opportunities

Bioinformatic training presents a variety of challenges [23, 24], and the results of this survey highlight several of note. First, there is considerable interest in a broad array of bioinformatics topics: even the topic with the lowest interest, phylogenetic analyses, received an interest score of 4 or 5 from 25% of respondents. Given these diverse interests of researchers, it is unlikely that any single person would have the expertise to provide training for all topics listed in this survey. This reinforces the necessity for partnerships outside of the library, with other intramural or extramural units with appropriate levels of expertise. The pace of change in bioinformatics is considerably rapid, as evidenced by interest in transcriptomics and genomic pipelines, both of which are characterized by tools that have only recently become accessible to most researchers. It is thus imperative for bioinformatics trainers to keep abreast of the latest trends and available training resources. Finally, the diverse expertise of health sciences researchers, from zero to extensive bioinformatics training, presents challenges when developing training opportunities. Addressing this diversity effectively requires careful consideration of training expectations–often requiring multiple, separate sessions in order to meet the needs of novices and those researchers already possessing some proficiency in bioinformatics. The interdisciplinary nature of bioinformatics argues for a collaborative approach to address the challenges presented by the diverse needs and backgrounds of researchers interested in bioinformatics.

## Conclusion

This assessment of bioinformatic training needs, and initial steps to address these needs, should serve as guidance to other libraries looking to establish or improve bioinformatics support programs. There is clear interest in bioinformatic training in the health sciences, and libraries are poised to support navigation of various biological data sources and, where appropriate, analytical treatment of said bioinformation during the research process. Assessing bioinformatic needs is critical in the development of a service program [10], and it is also important for existing programs, in order to keep up with changing trends in health science research [9]. While this and previous surveys [8–12] provide templates for assessing training needs, additional areas of interest, such as data management, general statistics, and cloud computing, could be included in future assessments. Addressing these needs could follow the three general approaches taken at the University of Arizona Health Sciences Library: (1) Develop workshops on topics of interest that fall within library staff areas of expertise. When the needs are beyond the library staff capacity, (2) partner with other campus units to provide bioinformatics support or (3) organize training from extramural resources. The first of these approaches, programming delivered by library staff with appropriate expertise, also acts as a point of contact with researchers, providing opportunities which may result in additional, in-depth collaboration between the library and health scientists.

**Fig 4:**
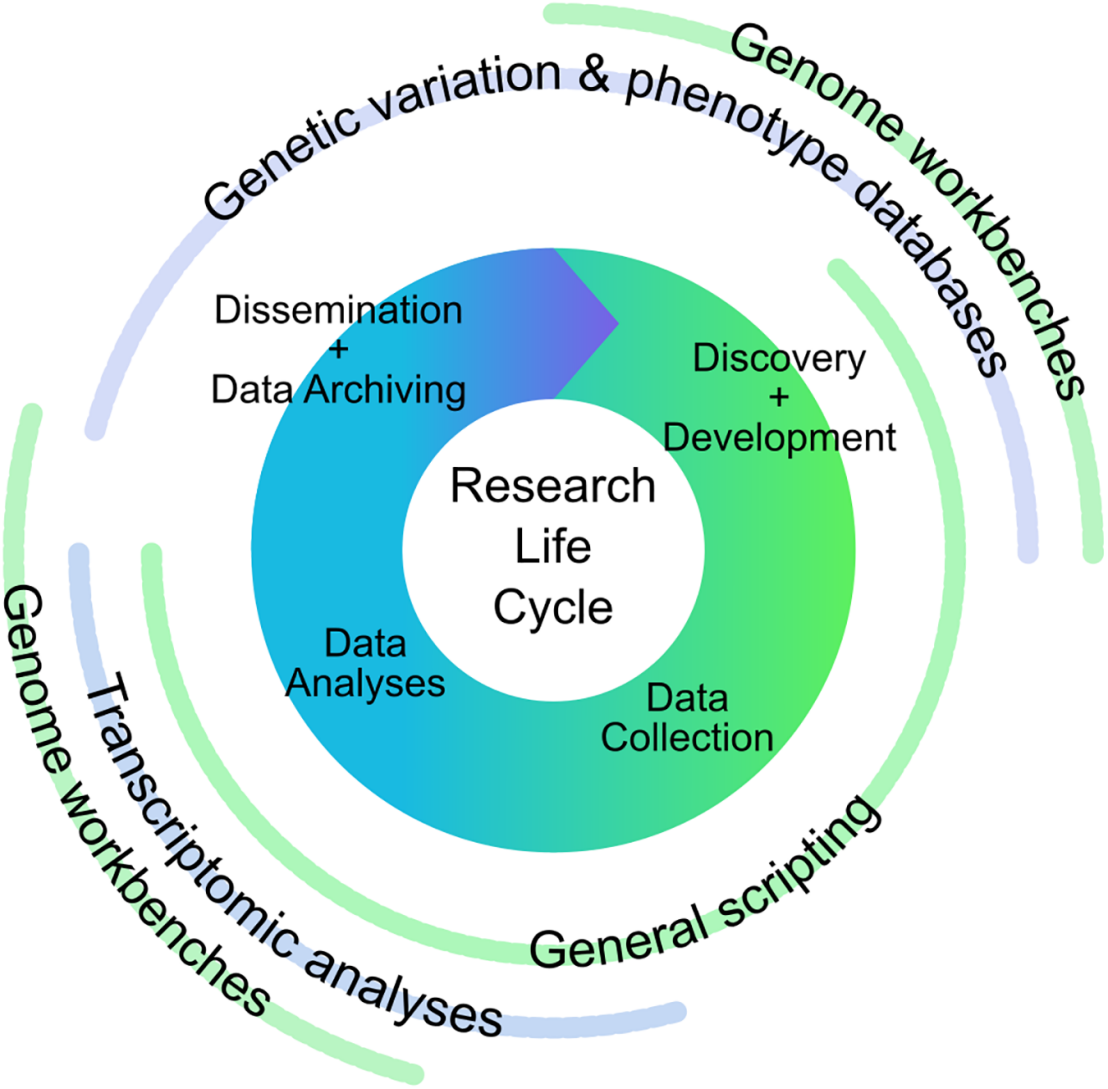
Points of contact in the research life cycle between researchers and library personnel. The four topics garnering the most interest (transcriptomic analyses, genome workbenches, general scripting, and variation databases) are shown at the points in a stylized research life cycle where experienced library staff may support health science researchers.

Library support for bioinformatics represents an area of overlap between information literacy and computational literacy, and it highlights the evolution of libraries' roles in the research life cycle (Fig 4). The idea that libraries are about more than bibliographic information is not new [25], and researchers are looking for assistance in ways to access *data*, in addition to *information*. As a growing number of databases have Application Programming Interfaces (APIs) for downloading data, even access to some data requires familiarity with writing computer code. Supporting access to data, as well as supporting tools for analyses of those data, is a rich opportunity for libraries to become more involved in research. Additional investment in subject-specialists and experience in the research process would considerably facilitate further integration of libraries into research in the health sciences and beyond.

## Supporting information

S1 File Bioinformatic training needs assessment survey. (PDF)

S1 Fig Number of completed surveys over total survey time. Vertical bars indicate 12-hour intervals immediately succeeding the three survey e-mail notifications (one initial invitation and two reminders).
(SVG)
