# Supplementary material for: Bioinformatic training needs at a health sciences campus
Source: PLoS One. 2017 Jun 14;12(6):e0179581. doi: 10.1371/journal.pone.0179581 (PMC5470718; doi:10.1371/journal.pone.0179581)
Supplement: S1 File — (PDF) [file pone.0179581.s001.pdf]

S1 File. Bioinformatic training needs assessment survey.

Bioinformatic Needs Assessment

To serve the needs of the health sciences research community, the Arizona Health Sciences Library now has a full-time Bioinformationist on staff. The purpose of this survey is to identify the primary bioinformatic needs of the community in order to tailor the services offered by the Bioinformationist. Participation is entirely optional, but your feedback will shape the support and training opportunities that are ultimately offered. Responses are anonymous, although there will be an opportunity to provide contact information for a bioinformatics mailing list. The results of this survey, void of any personal identifiers, may be used in future publications. The survey should take 5-8 minutes to complete.

Training Opportunities

How interested are you in training opportunities on the following topics?

	Not interested				Very interested
NCBI searching (Entrez system, Entrez E-Utilities for high-throughput queries)	❍	❍	❍	❍	❍
Sequence similarity tools (BLAST, BLASTn, PSI-BLAST)	❍	❍	❍	❍	❍
Nucleotide databases (GenBank, RefSeq)	❍	❍	❍	❍	❍
Genetic variation & phenotypes (NCBI Variation viewer, dbVar, dbSNP)	❍	❍	❍	❍	❍
Genome workbenches (NCBI Genome Workbench, Ensembl Genome Browser, UCSC Genome Browser)	❍	❍	❍	❍	❍
Protein 3-D structure (Cn3D, VAST, I-TASSER, Robetta)	❍	❍	❍	❍	❍
Transcriptome assembly and analysis (including splice variants and differential gene expression) (Trinity, DAVID)	❍	❍	❍	❍	❍
Genomic pipelines (e.g. scans for selection, high throughput annotation) (Galaxy, Bioconductor)	❍	❍	❍	❍	❍
Multiple sequence alignment (MAFFT, CLUSTAL, MUSCLE)	❍	❍	❍	❍	❍
Phylogenetic inference (RAxML, BEAST, MrBayes, Mesquite)	❍	❍	❍	❍	❍
General scripting (R, python)	❍	❍	❍	❍	❍
NIH compliance with Public Access Policy or Genomic Data Sharing Policy	❍	❍	❍	❍	❍

List any additional topics for which you would like training opportunities to be offered:

Which method(s) of training and support do you prefer? (check all that apply)

❑ Hands-on workshop
❑ Lecture
❑ One-on-one session
❑ Group session (i.e. training for lab or research group)
❑ On-line tutorial
❑ Webinar
❑ Other (please specify): ____________________
❑ N/A

How often do you use the following library resources?

	Never	Yearly	Monthly	Weekly	Daily
Physical resources (library workspace, print material)	❍	❍	❍	❍	❍
Personnel resources (librarians, analysts)	❍	❍	❍	❍	❍
Online resouces (e.g. Google Scholar, Embase, Web of Science)	❍	❍	❍	❍	❍

Your Details

Position (choose the one option that best describes your position at the University of Arizona)

❍ Faculty
❍ Staff
❍ Post-doc
❍ Graduate student
❍ Visiting scholar
❍ Appointed professional
❍ Other (please specify) ____________________

College ____________________

Department ____________________

How long have you been at the University of Arizona?

Your Research

Which organism(s) does your research actively involve?

Which, if any, molecules are actively investigated in your research? (Check all that apply)
- ❑ DNA
- ❑ RNA
- ❑ Proteins
- ❑ Other (please specify): ____________________
- ❑ N/A

If you use high-throughput technologies in your research, which platform(s) do you commonly use? (Check all that apply)
- ❑ Microarray
- ❑ Next-generation Sequencing
- ❑ qPCR array
- ❑ Other (please specify): ____________________
- ❑ N/A

If you use next-generation sequencing technologies in your research, which platform(s) do you commonly use? (Check all that apply)
- ❍ Illumina
- ❍ PacBio
- ❍ IonTorrent
- ❍ 454
- ❍ SOLiD
- ❍ Other (please specify): ____________________
- ❍ N/A

Funding

What are your major sources of funding?
- ❑ NIH
- ❑ NSF
- ❑ Other (please specify): ____________________

Are you required to submit copies of publications to PubMed Central? (Any direct funding from NIH grants awarded in 2008 or beyond requires peer-reviewed publications to be submitted to PubMed Central)
- ❍ Yes
- ❍ No
- ❍ I don't know

Are you required to share genomic research data? (Any genomic data resulting from NIH funding from grants or contract submitted on or after January 25, 2015 requires adherence to the NIH Genomic Data Sharing Policy)
- ❍ Yes
- ❍ No
- ❍ I don't know
